# Prevalence and Factors Associated with Tobacco Use Among Adults Attending Selected Healthcare Facilities in the OR Tambo District, Rural Eastern Cape Province, South Africa

**DOI:** 10.3390/ijerph23040452

**Published:** 2026-04-01

**Authors:** Bayebande Christelle Muke, Guillermo-Alfredo Pulido-Estrada

**Affiliations:** School of Public Health, Faculty of Medicine and Health Sciences, Walter Sisulu University, Mthatha 5100, South Africa; pulido762@yahoo.com

**Keywords:** tobacco use, prevalence, associated factors, rural Eastern Cape, South Africa, alcohol consumption, education, gender disparities

## Abstract

**Highlights:**

**Public health relevance—How does this work relate to a public health issue?**

- Tobacco use remains a major preventable health problem in rural South Africa and contributes to chronic disease and early death.
- This study addresses limited local evidence on tobacco use in adults attending healthcare facilities in the rural OR Tambo District.

**Public health significance—Why is this work of significance to public health?**

- More than one in four adults in the study reported current tobacco use, with much higher use among men.
- Tobacco use was strongly associated with alcohol consumption and lower education, identifying groups at higher risk.

**Public health implications—What are the key implications or messages for practitioners, policy makers and/or researchers in public health?**

- Primary healthcare services should strengthen routine screening and integrated cessation support for both tobacco and alcohol use.
- Public health policies and programmes in rural communities should prioritise male-focused, education-sensitive prevention, and cessation strategies.

**Abstract:**

Tobacco use is a primary global public health concern. In South Africa, particularly in the rural Eastern Cape, there is a paucity of comprehensive information about the burden of tobacco use and its associated factors. This study aimed to determine the prevalence of tobacco use and identify associated factors among adults seeking care at healthcare facilities in the OR Tambo District. A quantitative cross-sectional study was conducted among 246 adults (≥18 years) using a three-stage random sampling of sub-districts, clinics, and participants. Data were collected through structured interviews using the WHO STEPS questionnaire. Descriptive statistics, Chi-square tests, t-tests, and logistic regression analyses were applied. Overall, 27.2% of participants reported current tobacco use, with prevalence substantially higher among men (70.4%) than women (21.9%). Multivariate logistic regression confirmed male gender (AOR = 8.14, *p* < 0.001) and alcohol consumption (AOR = 19.5, *p* < 0.0001) as independent predictors of tobacco use. Higher education reduced smoking risk significantly (secondary education: AOR = 0.15; *p* = 0.036; tertiary education: AOR = 0.07; *p* = 0.014). Tobacco use remains highly prevalent in the rural Eastern Cape. Targeted interventions should focus on men, integrate tobacco–alcohol cessation programs, and expand education-based prevention strategies to reduce the burden of tobacco use in rural communities.

## 1. Introduction

Tobacco use remains one of the leading preventable causes of death globally, accounting for more than 8 million deaths each year, including approximately 1.3 million deaths attributable to second-hand smoke exposure [1]. Tobacco smoking contributes substantially to the global burden of non-communicable diseases, including cardiovascular disease, cancer, and chronic respiratory diseases [1,2]. Globally, tobacco use is unevenly distributed, with the highest prevalence occurring in low- and middle-income countries where nearly 80% of the world’s smokers reside [1]. Socioeconomic inequality, aggressive tobacco industry marketing, and weak regulatory enforcement contribute to sustained tobacco consumption in these settings.

In Sub-Saharan Africa, tobacco use has historically been lower than in other regions, but prevalence has been increasing in recent decades due to population growth, urbanisation, and expanding tobacco markets [3]. Current evidence indicates that adult smoking prevalence in many African countries ranges between 15% and 25%, with considerably higher prevalence among men compared with women [4,5]. These trends raise major public health concerns given the growing burden of non-communicable diseases in the region.

In South Africa, tobacco use remains a major public health challenge. According to the Global Adult Tobacco Survey (GATS) conducted in 2021, approximately 29.4% of adults aged 15 years and older reported current tobacco use, with prevalence markedly higher among men (41.7%) than among women (17.9%) [6]. Tobacco use contributes substantially to South Africa’s burden of cardiovascular disease, cancer, tuberculosis, and chronic respiratory disease [7,8]. Despite the introduction of strong tobacco control policies including advertising bans, smoke-free legislation, and excise taxation, tobacco use continues to persist in socioeconomically disadvantaged populations.

Tobacco use often disproportionately affects rural populations worldwide [9]. Studies from the United States, and several low- and middle-income countries have consistently reported higher smoking prevalence in rural areas compared with urban populations [10]. These disparities are commonly attributed to lower educational attainment, higher poverty rates, cultural acceptance of smoking, and limited access to cessation services [11,12].

Similar patterns have been documented in South Africa. Rural communities frequently face structural disadvantages including high unemployment, limited healthcare infrastructure, and restricted access to health promotion programmes [6,13]. These conditions can contribute to higher tobacco use and reduced cessation success. Research conducted in rural provinces such as the Eastern Cape and Limpopo indicates that tobacco use is often embedded within social norms and coping strategies related to economic stress and social marginalisation [5,10]. Furthermore, tobacco cessation services remain scarce in many rural clinics, limiting opportunities for behavioural counselling, pharmacological support and hinder the achievement of Sustainable Development Goal 3 [14,15].

Despite these challenges, relatively few studies have examined tobacco use patterns within rural primary healthcare populations in South Africa. Most available data originate from national surveys that may obscure local variations within deeply rural districts. Understanding tobacco use patterns in such settings is essential for designing context-specific public health interventions.

## 2. Materials and Methods

### 2.1. Study Design

The study employed a quantitative cross-sectional design to determine the prevalence of tobacco use and identify associated factors among adults attending healthcare facilities in the rural Eastern Cape Province of South Africa. This design was appropriate for simultaneously assessing exposure (tobacco use) and potential associated factors at a single point in time.

#### 2.1.1. Study

Population and Sampling: The study population comprised adults aged 18 years and older attending selected primary healthcare facilities in the OR Tambo District of the Eastern Cape Province. The research was conducted in four primary healthcare facilities located within two randomly selected sub-districts, King Sabata Dalindyebo (KSD) and Mhlontlo. A three-stage sampling strategy was applied: (1) two sub-districts were randomly selected; (2) two clinics per sub-district were chosen; (3) eligible adults were randomly recruited until the sample size was reached. An estimated prevalence (P) of 20% (0.20) was used [6], with a 5% margin of error (0.05) at a 95% confidence level, yielding a required sample size of 246 participants.

#### 2.1.2. Inclusion and Exclusion

Inclusion criteria: Adults aged 18 years and older attending the selected clinics during the study period and willing to provide written informed consent. Exclusion criteria: Patients who were too ill to participate or who declined consent.

### 2.2. Data Collection

Data were collected using the WHO STEPS questionnaire (World Health Organization 20 Avenue Appia, Geneva, Switzerland), a standardized and validated tool developed by the World Health Organization to assess risk factors for non-communicable diseases, including tobacco use [16]. The instrument was adapted to the study context, translated into the local language (Xhosa), and reviewed for cultural appropriateness. The researcher administered the questionnaires through face-to-face structured interviews in a private area from 6 June 2025 to 1 September 2025.

### 2.3. Data Management and Statistical Analysis

Data were entered into a secure electronic database with double-entry verification. Cleaning included checks for missing values, inconsistencies, and outliers. Descriptive statistics summarized variables (frequencies, percentages, means, medians). Bivariate analyses used Chi-square or Fisher’s exact tests for associations. Multivariate logistic regression identified independent predictors, reported as adjusted odds ratios (AORs) with 95% confidence intervals. Analyses were conducted using R version 4.5.1 (The R Foundation for Statistical Computing, based in Vienna, Austria), with *p* < 0.05 considered significant.

### 2.4. Ethics and Legal Considerations

The study adhered to the ethical principles of respect for participants, beneficence, non-maleficence, and justice outlined in the Declaration of Helsinki. Ethical approval was obtained from the Human Research Ethics Committee of the Faculty of Medicine and Health Sciences, Walter Sisulu University (Reference: WSU HREC033/2025). Permission was granted by the Eastern Cape Department of Health and the OR Tambo District Office (Reference: EC_202505_017). All participants provided written informed consent, with assurances of confidentiality and voluntary participation.

## 3. Results

This section is divided by subheadings to provide a concise and precise description of the experimental results, their interpretation, as well as the experimental conclusions that can be drawn.

### 3.1. Participant Characteristics

A total of 246 adult records were analysed, with a mean age of approximately 44 years (median 44, IQR 32–55). Most participants were females (72.8%), and the sample was stratified by age groups: 18–35 years (27.2%), 36–50 years (37.8%), and >50 years (31.3%).

### 3.2. Prevalence of Tobacco Use

In this study, 67 out of 246 participants reported current tobacco use, yielding a prevalence rate of 27.2%. Prevalence varied by age and gender (Table 1). Among participants aged 18–35 years, 29.9% were smokers; in the 36–50 years group, 22.6%; and in the >50 years group, 33.8%. Men reported higher rates (70.4%) than women (21.9%).

#### Prevalence of Tobacco Users Stratified by Age and Gender

Gender differences were pronounced. Across all age groups, men were substantially more likely to smoke than women. For example, in the 18–35 years group, 57.1% of men were smokers compared with 24.6% of women; in the 36–50 years group, nearly 9 in 10 men (88.9%) smoked compared with 14.9% of women; and among participants over 50 years, 63.6% of men and 28.4% of women reported current tobacco use.

### 3.3. Demographic and Socioeconomic Characteristics and Associated Factors

Table 2 presents the distribution of tobacco use across key demographic and socioeconomic variables. Marked gender differences were observed, with 70.4% of men reporting current smoking compared with only 21.9% of women. Smoking prevalence varied modestly across age categories—from 21.9% to 33.3%—without a clear upward or downward trend. Marital status showed minimal variation, with smoking reported by 28.9% of married participants and 23.8% of single participants. Education demonstrated a strong inverse gradient: smoking was highest among those with no formal education (75.0%) and primary education (38.1%), decreasing substantially among those with secondary (23.4%) and tertiary education (9.3%). Employment status also showed disparities, with higher smoking prevalence among unemployed (36.2%) and self-employed individuals (33.3%) compared with the formally employed (14.9%). A similar socioeconomic pattern was evident for income, where smoking was more common among lower-income groups (<R1000 and R1000–R5500: 30.1–32.7%) and considerably lower among participants earning R5001–R10,000 (5.9%) or more than R10,000 (10.7%). Alcohol consumption emerged as a strong behavioural correlate: 78.0% of alcohol users were smokers compared with only 14.3% of non-drinkers. When age was analysed as a continuous variable, no meaningful differences were found between smokers and non-smokers, reinforcing the absence of an association between age and tobacco use in this study population.

### 3.4. Bivariate Analysis

Table 3 shows that smoking was significantly associated with several demographic and socioeconomic factors. Gender differences were pronounced: males were far more likely to smoke (70.4%) than females (21.9%), indicating a clear gender disparity in tobacco use. Although smoking prevalence was highest among participants aged 51 years and older (33.3%) and lowest in the 36–50-year age group (21.9%), these differences were not statistically significant, suggesting that age is not a primary determinant of smoking behaviour in this population. Similarly, marital status was not significantly related to smoking, with only small differences between married (28.9%) and single (23.8%) participants. In contrast, education level showed a strong inverse association with smoking, with prevalence decreasing from 75.0% among those with no formal education to 9.3% among those with tertiary education, highlighting education as a protective factor. Employment status was also significantly associated with tobacco use, as unemployed (36.2%) and self-employed participants (33.3%) were more likely to smoke than those who were formally employed (14.9%). A similar pattern was observed for income: smoking was more prevalent among lower-income groups (30.1–32.7%), whereas participants earning R5001–R10,000 had the lowest prevalence (5.9%), suggesting an important role of socioeconomic status. Alcohol use emerged as a particularly strong correlate, with 78.0% of alcohol consumers reporting smoking compared to only 14.3% of non-drinkers, indicating substantial clustering of these behaviours. Overall, these findings indicate that gender, education, employment, income, and alcohol consumption are key factors associated with tobacco use in this setting, whereas age and marital status do not show statistically significant associations despite some observable differences in prevalence.

### 3.5. Multivariate Analysis

The multivariate logistic regression analysis (Table 4) identified several variables as significant independent predictors of tobacco use. Gender remained a strong determinant, with males exhibiting more than eight times the odds of smoking compared with females (AOR = 8.14, 95% CI: 2.42–30.10, *p* = 0.0009), highlighting a substantial gender disparity in tobacco use within this population. Education level showed a clear protective effect: individuals with secondary education had an 85% lower likelihood of smoking compared with those with no schooling (AOR = 0.15, 95% CI: 0.02–0.87, *p* = 0.036), while tertiary education was associated with a 93% reduction in smoking odds (AOR = 0.07, 95% CI: 0.01–0.55, *p* = 0.0149), demonstrating a strong inverse relationship between educational attainment and tobacco use. Alcohol consumption emerged as the most powerful predictor; participants who consumed alcohol had nearly twenty times higher odds of smoking than those who abstained (AOR = 19.50, 95% CI: 8.17–51.20, *p* < 0.0001), indicating a close behavioural link and clustering of these risk factors. Other demographic and socioeconomic variables such as age, marital status, employment, and income did not show significant associations in the adjusted model, suggesting that their apparent effects in descriptive analyses were largely explained by gender, education, and alcohol use. Overall, the multivariate findings indicate that being male and consuming alcohol are the strongest risk factors for tobacco use, while higher levels of education provide significant protection.

## 4. Discussion

The markedly higher prevalence of tobacco use among men observed in this study reflects well-established gender disparities in smoking patterns across South Africa and other Sub-Saharan African countries, where smoking is frequently associated with masculinity, adulthood, and social identity [17]. In many rural communities, smoking may be perceived as a marker of resilience, adulthood, or social status, particularly among men facing economic hardship or limited employment opportunities [18]. Such social norms can reinforce tobacco use while simultaneously discouraging cessation attempts. Conversely, smoking prevalence among women is typically lower, which may partly reflect social stigma associated with female smoking in many African societies. Qualitative research conducted in South Africa suggests that women may under-report tobacco use due to cultural expectations surrounding gender roles and socially acceptable behaviour [5]. These gendered perceptions of smoking may therefore influence both the actual tobacco use patterns and reporting behaviour in survey-based studies.

Education emerged as a strong protective factor against tobacco use in this study, consistent with previous evidence indicating that higher educational attainment improves health literacy, risk perception, and access to information regarding smoking cessation resources [5,8]. Individuals with higher levels of education may be more aware of the long-term health risks associated with tobacco use and better equipped to avoid or quit smoking. Conversely, limited schooling combined with socioeconomic stressors may increase vulnerability to tobacco use, as smoking may be used as a coping mechanism for financial strain or psychosocial stress [19].

The strong association between alcohol consumption and tobacco use observed in this study indicates a clustering of behavioural risk factors. Similar findings have been reported in South Africa and other low- and middle-income countries, where tobacco and alcohol use often co-occur in shared social environments such as taverns, informal gatherings, and social events [20]. In such contexts, smoking and alcohol consumption may reinforce one another through social normalization and immediate pleasurable effects [21]. From a behavioural perspective, this co-use may also be explained through mechanisms of operant conditioning, where the short-term relief of stress or social bonding associated with these behaviours outweighs concerns about long-term health consequences.

Interestingly, the absence of independent associations between tobacco use and employment or income in the multivariate model contrasts with findings from several studies that identify socioeconomic status as a major determinant of smoking behaviour [5,9]. Previous research has shown that individuals with lower socioeconomic status often experience higher smoking prevalence due to financial stress, limited health literacy, and reduced access to cessation services [22]. In the present study, the apparent influence of employment and income may be largely mediated through education and alcohol consumption, which may represent more proximal determinants of smoking behaviour. It is also possible that the relatively modest sample size limited the statistical power to detect smaller socioeconomic effects.

The prevalence of tobacco use observed in this study (27.2%) closely aligns with national estimates reported in the 2021 Global Adult Tobacco Survey, which reported a prevalence of approximately 29.4% among South African adults [8]. These findings are also consistent with studies conducted in other rural South African settings and across Sub-Saharan Africa, where tobacco use remains prevalent in contexts characterized by socioeconomic disadvantage, weak enforcement of tobacco control policies, and limited access to cessation services [11,12]. The strong co-occurrence of alcohol and tobacco use further supports evidence suggesting that integrated interventions targeting multiple behavioural risk factors are more effective than single-behaviour approaches.

Taken together, these findings highlight an urgent need for context-specific, gender-sensitive tobacco control strategies in rural Eastern Cape communities. Priority interventions should include male-focused cessation programmes that address cultural norms linking smoking with masculinity, combined with tailored behavioural counselling and access to pharmacological cessation support. Strengthening education and health literacy initiatives is also critical. Culturally appropriate health education campaigns delivered in local languages through schools, community structures, and primary healthcare clinics could increase the awareness of tobacco-related health risks and the benefits of cessation while helping prevent smoking initiation among younger populations. Integrating routine tobacco and alcohol screening within primary healthcare services may further improve the early identification of high-risk individuals and facilitate combined behavioural interventions. Finally, strengthening the enforcement of existing tobacco control policies, including smoke-free legislation and restrictions on tobacco marketing, particularly in rural areas, will be essential for reducing tobacco availability and social acceptability. Collaboration between health authorities, local government, and community leaders will be critical to ensure the sustainability and effectiveness of these interventions.

This study has several strengths. It used a standardized WHO STEPS instrument and multi-stage random sampling to recruit participants from multiple clinics, enhancing internal validity and representativeness within the district. It also provides rare, district-level data on tobacco use in a rural Eastern Cape population. However, limitations must be acknowledged. First, the cross-sectional design precludes causal inference; associations between factors such as alcohol use and smoking cannot be interpreted as cause–effect relationships. Second, smoking status and other behavioural variables were self-reported and may be affected by social desirability bias, particularly among women. Third, the facility-based sample may not fully represent all adults in the rural communities, especially those who do not access formal healthcare services. Fourth, the sample size, although adequate for primary analyses, may have limited the ability to detect modest associations for some variables, such as income and employment. Finally, unmeasured confounders, including mental health status and stress, may have influenced both tobacco use and the explanatory variables.

## 5. Conclusions

This study highlights a significant burden of tobacco use (27.2%) among adults in rural Eastern Cape, with male gender, low education, and alcohol consumption as key risk factors. Higher education levels emerged as a protective factor, underscoring the potential of education-based interventions. The findings align with national and global trends [1,8], emphasizing the need for multifaceted, context-specific strategies to address tobacco use in resource-limited rural settings. By identifying high-risk groups and protective factors, this study provides a foundation for evidence-based interventions to reduce the health and economic burdens of tobacco use in the OR Tambo District.

## Figures and Tables

**Table 1:** Prevalence of tobacco users by age and gender.

Age Group	Total (n = 246)	Gender	Non-Smokers n (%)	Smokers n (%)
18–35	65	female	49 (75.4)	16 (24.6)
	7	male	3 (42.9)	4 (57.1)
36–50	87	female	74 (85.1)	13 (14.9)
	9	male	1 (11.1)	8 (88.9)
>50	67	female	48 (71.6)	19 (28.4)
	11	male	4 (36.4)	7 (63.6)
Total	246		179 (72.8)	67 (27.2)

**Table 2:** Demographic and socioeconomic characteristics of the study population by smoking status.

Variable	Non-Smokers n (%)	Smokers n (%)
Gender		
Female	171 (78.1)	48 (21.9)
Male	8 (29.6)	19 (70.4)
Age category		
18–35	52 (72.2)	21 (27.8)
36–50	75 (78.1)	21 (21.9)
>50	52 (66.7)	26 (33.3)
Marital status		
Single	61 (76.2)	19 (23.8)
Married	118 (71.1)	48 (28.9)
Education category		
None	3 (25)	9 (75)
Any formal education	124 (75)	58 (25)
Employment status		
Unemployed	81 (45.25)	46 (36.2)
Employed/self	98 (54.75)	6 (33.3)
Income bracket		
Less than R1000	72 (69.9)	31 (30.1)
R1000–R5500	66 (67.3)	32 (32.7)
R5001–R10,000	16 (94.1)	1 (5.9)
More than R10,000	25 (89.3)	3 (10.7)
Alcohol consumption		
No	168 (85.7)	28 (14.3)
Yes	11 (22)	39 (78)

**Table 3:** Bivariate analysis comparing demographic and socioeconomic factors with smoking status.

Variable	Non-Smokers n (%)	Smokers n (%)	*p*-Value
Gender			<0.0001
Female	171 (78.1)	48 (21.9)	
Male	8 (29.6)	19 (70.4)	
Age category			0.238
0–35	52 (72.2)	20 (27.8)	
36–50	75 (78.1)	21 (21.9)	
>50	52 (66.7)	26 (33.3)	
Marital status			0.484
Single	61 (76.2)	19 (23.8)	
Married	118 (71.1)	48 (28.9)	
Education category			<0.0001
None	3 (25)	9 (75)	
Primary	39 (61.9)	24 (38.1)	
Secondary	98 (76.6)	30 (23.4)	
Tertiary	39 (90.7)	4 (9.3)	
Employment status			0.001
Unemployed	81 (63.8)	46 (36.2)	
Self-Employed	12 (66.7)	6 (33.3)	
Employed	86 (85.1)	15 (14.9)	
Income bracket			0.021
Less than R1000	72 (69.9)	31 (30.1)	
R1000–R5500	66 (67.3)	32 (32.7)	
R5001–R10,000	16 (94.1)	1 (5.9)	
More than R10,000	25 (89.3)	3 (10.7)	
Alcohol consumption			<0.0001
No	168 (85.7)	28 (14.3)	
Yes	11 (22)	39 (78)	

**Table 4:** Logistic regression to determine the risk factors associated with tobacco use.

Variable	Adjusted Odds Ratio (AOR)	95% CI	*p*-Value
Gender			
Female	(Reference)		
Male	8.14	[2.42–30.10]	<0.001
Income category (ZAR)			
Less than 1000	(Reference)		
1000–5500	1.08	[0.43–2.66]	0.874
5501–10,000	0.15	[0.00–1.32]	0.144
Above 10,000	0.82	[0.11–4.93]	0.838
Employment status			
Unemployed	(Reference)		
Self-employed	1.1	[0.26–4.13]	0.889
Employed	0.42	[0.13–1.21]	0.118
Education category			
None	(Reference)		
Primary	0.26	[0.03–1.58]	0.149
Secondary	0.15	[0.02–0.86]	0.036
Tertiary	0.07	[0.00–0.56]	0.014
Alcohol consumption			
No	(Reference)		
Yes	19.5	[8.17–51.2]	<0.0001

## Data Availability

Study data are available from the corresponding author and/or the Eastern Cape Department of Health on reasonable request, subject to institutional and ethical regulations.

## Abbreviations

The following abbreviations are used in this manuscript:

AORs	Adjusted Odds Ratio
CI	Confidence Interval
COPD	Chronic Obstructive Pulmonary Disease
FCTC	Framework Convention on Tobacco Control
GATS	Global Adult Tobacco Survey
GBD	Global Burden of Disease
HBM	Health Belief Model
KSD	King Sabata Dalindyebo
LMICs	Low- and Middle-Income Countries
MPH	Master of Public Health
SADHS	South African Demographic and Health Survey
SDG	Sustainable Development Goal
SLT	Social Learning Theory
TPB	Theory of Planned Behaviour
TTM	Transtheoretical Model
WHO	World Health Organization
